# Therapeutic Interventions in Rat Models of Preterm Hypoxic Ischemic Injury: Effects of Hypothermia, Caffeine, and the Influence of Sex

**DOI:** 10.3390/life12101514

**Published:** 2022-09-28

**Authors:** Ruth McLeod, Ted Rosenkrantz, Roslyn Holly Fitch

**Affiliations:** 1 Psychological Science, University of Connecticut, Storrs, CT 06269, USA; 2 School of Medicine, University of Connecticut, Farmington, CT 06030, USA

**Keywords:** preterm, hypoxia, ischemia, animal models, caffeine, hypothermia, sex differences

## Abstract

Infants born prematurely have an increased risk of experiencing brain injury, specifically injury caused by Hypoxia Ischemia (HI). There is no approved treatment for preterm infants, in contrast to term infants that experience Hypoxic Ischemic Encephalopathy (HIE) and can be treated with hypothermia. Given this increased risk and lack of approved treatment, it is imperative to explore and model potential treatments in animal models of preterm injury. Hypothermia is one potential treatment, though cooling to current clinical standards has been found to be detrimental for preterm infants. However, mild hypothermia may prove useful. Caffeine is another treatment that is already used in preterm infants to treat apnea of prematurity, and has shown neuroprotective effects. Both of these treatments show sex differences in behavioral outcomes and neuroprotective effects, which are critical to explore when working to translate from animal to human. The effects and research history of hypothermia, caffeine and how sex affects these treatment outcomes will be explored further in this review article.

## 1. Introduction

Approximately 11–12% of newborns in the US and 11% globally are premature, meaning born at less than 37 gestational weeks (GW) of prenatal development (aka preterm) [1,2,3]. These infants have increased mortality risk and are vulnerable to medical conditions that compromise cerebral oxygen supply and brain function, as well as impair long-term outcomes. Statistics indicate that more than 30% of premature infants have significant long-term disabilities [4,5]. Beyond stabilizing care, neonatologists have few medical options to protect surviving premature infants from long-term risks. There are no approved preterm neuroprotective treatments available, even for infants with severe intra-cranial bleeding or other forms of major encephalopathy. The high rates of premature birth, poor long-term outcomes, and lack of available treatments combine to make preterm brain injury a major health priority.

When cellular oxygen is reduced by hypoxia, ATP failure associated with inefficient anaerobic metabolism restricts neuronal activity. Complete oxygen restriction following ischemia or hypoxia-ischemia depletes high-energy metabolites even faster. This critical ATP loss leads to excess extracellular glutamate, prolonged neural depolarization, and elevated calcium and sodium influx. Maturity of glutamate receptors determines the rate of events [6,7], but eventually sodium over-load leads to cell swelling and necrotic cell death, while calcium over-load activates neuronal nitric oxide synthase (nNos) that accumulates and activates free radicals nitric oxide and peroxynitrate. Mitochondrial dysfunction and translocation of cytochrome-c from the mitochondria to the nucleus, caspase cleavage, chromatin condensation, and DNA fragmentation follow. This mitochondrial dysfunction also increases the levels of reactive oxygen species, that can contribute to cellular death [8]. Simultaneous activation of poly (ADP-ribose) polymerase-1 (Parp-1; a DNA repair enzyme) leads to release of apoptotic factors [9,10,11]. Both pathways cause an increase in inflammation, microglial activation, cell death, and tissue loss. Two potential treatments, hypothermia and caffeine may help mediate these effects.

Although the mechanisms of action for caffeine and hypothermia are not precisely known, therapeutic effects of hypothermia are thought to be mediated by a preservation of energy metabolism, reduction in cytotoxic edema and free radicals, and reduction in immune and inflammatory response as well as apoptotic cell death. Caffeine acts as an adenosine antagonist (specifically at A_2A_ and/or A_1_ receptors), and this interaction may attenuate calcium influx [12,13]. Caffeine may also reduce microglial activation [14] and/or reduce neuroinflammation either by decreasing the release of inflammatory proteins or blocking their action. [15].

Preterm brain injury and associated poor outcomes often originate in hypoxic-ischemic events (HI; decreased blood and/or oxygen supply) [16,17,18,19]. HI events often occur in the perinatal period. The associated preterm encephalopathies can follow from vascular fragility/immaturity and irregular blood pressure that together cause rupture and bleeding. Typical areas of injury involve the capillary-rich germinal matrix and/or peri-ventricular region (IVH hemorrhage). IVH still occurs in about 20–30% of severe preterm term infants despite now-routine antenatal corticosteroid use [16,17,18]. This injury particularly affects glial progenitors that are actively proliferating in the region, and are vulnerable to insult. Thus, intra-cranial hemorrhage in very preterm infants often damages white matter (e.g., motor tracts). This damage may be focal/cystic, or involve diffuse white matter loss (periventricular leukomalacia or PVL)—both are associated with cerebral palsy (CP) [17,18,20,21,22,23,24,25,26,27]. Perfusion failure/reperfusion injury can also result from falling blood pressure and capillary collapse, again with variable tissue loss. One of the most common causes of HI in the preterm brain is chronic hypoxemia secondary to respiratory insufficiency due to apnea, bradycardia and/or bronchopulmonary dysplasia (BPD). All of these conditions are very common in preterm infants. Resulting chronic low oxygen can cause diffuse brain injury in vascular “watershed” zones, and emergent PVL [19,21,28]. Although HI events are less common in late preterm infants, when they occur they typically lead to neurodegeneration associated with serious cognitive disabilities [17,18,29].

Other factors in the perinatal period that can affect susceptibility to HI include mothers experiencing chorioamnionitis, or preeclampsia, Preeclampsia is common in preterm mothers and leads to high blood pressure and protein in their urine. Infants born to preeclampsia mothers have a greater risk for periods of hypoxia and interventricular hemorrhage when compared to infants born prematurely but to mothers without preeclampsia [30]. Chorioamnionitis, an infection of the amniotic fluid and membranes surrounding the fetus, leads to an increase in inflammation for both the mother and infant fighting infection. Chorioamnionitis leads to an increased risk for PVL and IVH, and increased variability in brain oxygenation [31,32]. Events like placental abruption, which is more common in preterm delivery, increase instances of HIE in both term and preterm infants [33].

Poor outcomes associated with premature birth include blindness/deafness, cerebral palsy (CP), learning disability, ADHD, reduced neurodevelopmental scores (e.g., IQ and mental development index (MDI)) [34,35,36,37,38,39,40,41,42,43,44,45,46,47,48,49,50,51,52,53,54]. Specific statistics vary based on gestational age at birth, survival rates, medical complications, and criteria for study inclusion, but estimates suggest about 30% of surviving preterm infants (including those with and without HI events) experience cognitive delay or disability [55]. This rises to 40% for very preterm infants (<32 GW), and exceeds 50% for extremely preterm infants (<28 GW) [54]. Meta-analyses show that preterm children on average lose 10 IQ points, with specific difficulties in language acquisition and processing [45,56,57,58,59,60]. These language problems may relate to core underlying deficits in rapid auditory processing, such as the ability to process rapidly changing acoustic information in speech (e.g., /ba/ vs. /da/). Acoustic processing deficits are often seen in preterm infants, both with and without diagnosed brain injury [59,61,62,63,64]. Since these measures can be obtained pre-lingually (<1 year) they offer excellent prognostic value, and in fact early rapid acoustic processing scores are highly predictive of later language outcomes [61,63,64,65,66,67,68]. Importantly, these skills are not only affected under pathologic conditions, but predict language abilities in typically developing children as well [61,63,64,65,66,67,68,69]. This link provides a strong rationale for inclusion of rapid acoustic processing in animal models of early brain injury as a translational marker for language-relevant outcomes in clinical populations. Premature infants show other cognitive problems as well, including deficits in learning, memory, and executive functioning [69,70,71], poor performance on spatial memory tasks [41,72], and impairments in visual attention with heightened incidence of attention-deficit disorders [34,36,39,43,73,74,75,76,77,78,79].

## 2. Animal Models of Preterm Brain Injury

To explore behavioral outcomes following HI injury, animal models of induced insult have been developed in various species including sheep, piglets, and rodents [62,63,64,66,67,68,69,70,71,72,73,74]. Large-animal models offer key insight to cellular pathology of HI-induced white matter injury since small animals have less myelin compared to humans [80,81], but have drawbacks such as limitations in behavioral assessment. Overall, the most widely employed neonatal HI model is one adapted from Levine by Rice & Vannucci [82]. This model involves HI injury induced in rats on postnatal day (P) 7 by unilateral ligation/cauterization of a carotid artery (typically right) combined with a period of exposure to a hypoxic environment (45–180 min at 8% O^2^). For many years, P7 rats were thought to simulate *near*-*term* injury in human neonates (~38 GW;) [83] based on comparisons of peak brain growth across species [84,85]. With more precise mapping of cross-species neurodevelopmental markers, it is now apparent that a P7 rat equates more closely to a late preterm infant (32–34 GW) [86,87], with P7 HI in rats simulating late preterm brain injury [87,88]. Other authors argue that P7 corresponds to 36 GW, which is still earlier than previously thought, with P10 being roughly equivalent to a term infant [89]. P7 HI male rats exhibit reliable deficits in rapid auditory processing as juveniles and adults, and deficits are persistent and stable within subjects [90,91,92,93,94,95,96,97,98,99,100,101,102,103,104,105]. This follows other rodent models of language-related disabilities [106,107,108,109,110,111,112], and supports the idea that rapid acoustic processing deficits in animal models can serve as a biomarker of neural disruptions linked to language impairments [61,63,64,65,66,67,68]. Deficits on spatial and working memory tasks have also been validated in P7 HI rats (Morris Water Maze task, Non-Spatial Maze, delayed-match-to-place maze tasks) [93,96,97,113,114,115,116,117], as well as deficits in visual attention [114,118,119]. Perhaps based on this extensive literature, most pre-clinical neuroprotective HI studies use a P7 HI rat model (Rice-Vannucci) [88,104,105,120,121,122,123,124,125,126,127,128,129,130,131,132].

Large animal models (lambs and piglets) have been subjected to HI injury during fetal life. Typically, in utero umbilical/placental occlusion is used to simulate preterm-like brain injury, and as a platform to assess neuroprotective strategies [132,133,134,135,136]. Only a few studies have assessed preterm-like (<P6) HI injuries in rodent models [92,101,126,137,138,139,140]. Importantly, even fewer of these studies report comprehensive long-term behavioral assessments following preterm-like injury, or investigations of therapeutic intervention applicable to preterm infants.

## 3. Therapeutic Hypothermia for Neonatal Brain Injury

Currently, the most widely employed and extensively studied intervention for full-term infants with moderate to severe hypoxic ischemic encephalopathy (HIE)—a form of post-HI injury associated with birth complications (e.g., umbilical occlusion) is hypothermia (head or whole-body cooling) [141,142]. Using head-cap or cooling blankets, affected infants are cooled by about 4 °C (33.5 °C core temperature) within 6 h of birth, and maintained for 72 h before re-warming. These accepted parameters are the result of extensive, randomized multi-site cooling trials that measured mortality, morbidity, motor impairments/CP, sensory impairments, and cognitive outcomes [47,143,144,145]. Cooling of full-term infants with moderate to severe HIE has been shown to reduce mortality and incidence of major disability by about 25% (depending on inclusion criteria and outcome measures) [142] and improves mental/cognitive outcomes. Importantly, however, this therapy remains approved only for term infants, ≥ 36 GW. It should be noted that some clinicians are starting to use hypothermia at younger gestational ages. Despite being the most successful intervention used in term infants with HI (albeit with modest benefits), cooling has *not* been rigorously trialed or approved for <36 GW infants. Reports are limited to a handful of late preterm case studies, and one clinical trial that proposed using a term hypothermia regime (4 °C for 72 h) in preterm infants of 32–36 GW. However, this trial was halted after recruitment of only 4 infants, and reported 50% adverse outcome and 25% mortality [146,147,148] (NCT00620711). Another study has shown similar adverse outcomes from preterm hypothermia, but as there was no control group of uncooled preterm infants, it is difficult to say if the cooling truly caused more harm [18,33]. Concerns about use of cooling therapies in preterm infants center on possible deleterious complications like hypoglycemia and coagulopathy [18,148]. Indeed, the 72 h regimen optimized to term HI infants may *not* be effective in preterm infants. Most pediatric associations agree that it should not be used in preterm infants, outside of a research setting, due to potential deleterious outcomes [20]. However, premature infants might benefit from an abbreviated and milder temperature reduction as shown below in work by Smith [103,104].

**Figure 1:**
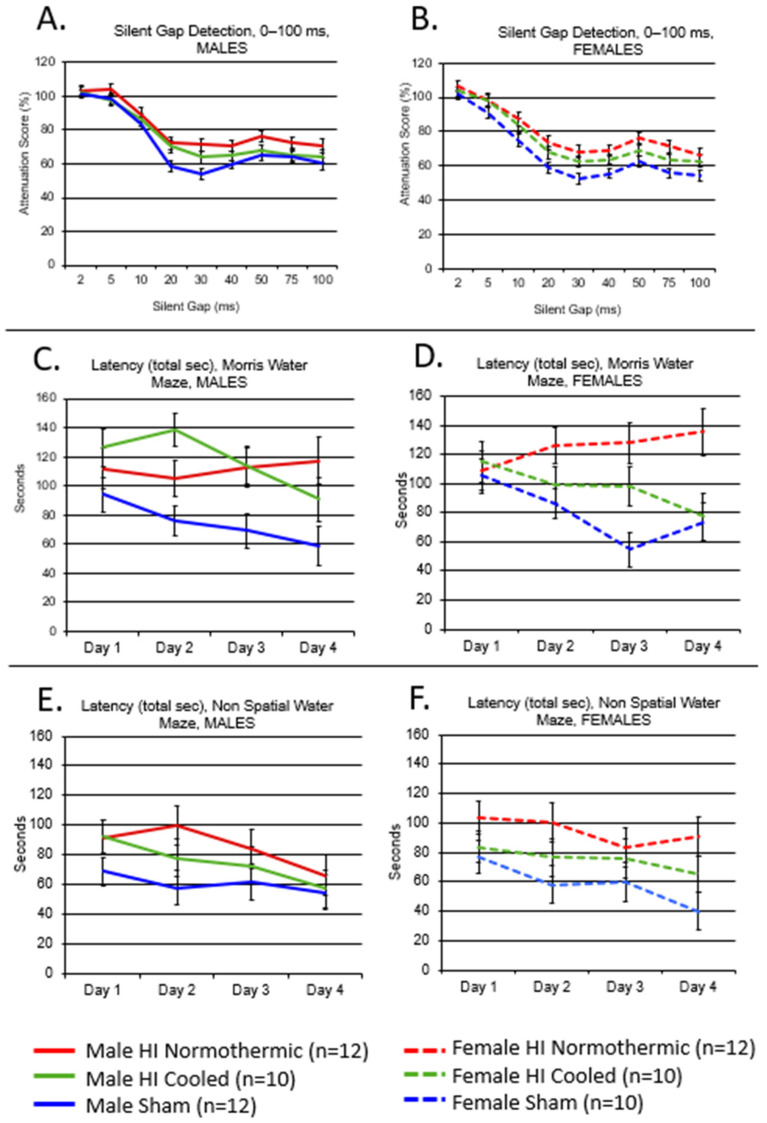
Cooling effects (Males and Females). Performance in P7 HI normothermic (red), HI cooled (green) and Sham normothermic (blue) male rats on: (**A**) silent gap 0–100 ms detection; (**C**) Morris Water Maze (MWM); and (**E**) Non-Spatial Water Maze (NSM). Performance in P7 HI normothermic (red), HI cooled (green) and Sham normothermic (blue) female rats on: (**B**) Silent Gap 0–100 ms detection; (**D**) Morris water maze (MWM); and (**F**) Non-Spatial Water Maze (NSM). Adapted with permission from Ref. [104]. 2015 by the authors.

Potter [149] conducted a study using a hypothermia treatment modeled after the approved therapy for term infants with moderate or severe brain injury (as defined by prolonged core temperature reduction of 4 °C). They reduced core body temperature of P6 rat pups by 4 °C for 5 h following induction of HI (human GA = 32–35 week). Matched littermates received HI followed by normothermic conditions, or sham treatment with comparable hypothermia or normothermia. Results showed that this cooling intervention was not only *ineffective*, but it was also deleterious to both sham and P6 HI rats as measured by behavioral and neuroanatomical outcomes [129,149]. Specifically, cooled sham males had worse scores on a Silent Gap acoustic task compared to normothermic sham males, and significantly worse scores on Non-Spatial Water Maze (*p* < 0.01) [149]. These findings have important implications for therapeutic intervention in at-risk preterm human populations, and certainly promote caution in the application of existing hypothermia protocols to at-risk preterm infants. However, they did successfully use a more modest form of temperature reduction in P7 HI rats, specifically a 1.5 °C temperature reduction for 2 h (Figure 1). This intervention—in contrast to the 4 °C/5 h regimen described above—was effective in mitigating some long-term behavioral deficits in both male and female rats with P7 HI injury and offered some protection from gross brain injury (Figure 1) [103,105]. Using a repeated measures ANOVA, cooled HI female and male rats showed improved performance compared to normothermic HI rats on a silent gap rapid auditory processing task (Females: *p* < 0.05, Males: *p* < 0.05) [104]. Cooled HI females and males also showed similar improvements in performance on the Morris Water Maze (females: *p* < 0.05, males: *p* < 0.05) compared to HI normothermic animals using a repeated measures ANOVA [104]. On the Non-spatial Water Maze a marginal treatment effect was seen in females, showing improved performance in the cooled HI group compared to normothermic HI animals (*p* = 0.08) [97]. Males on the same task did not show any overall treatment effects, but did show, through individual t-tests, normothermic rats doing significantly worse compared to shams (*p* = 0.05) [104].

## 4. Therapeutic Caffeine for Neonatal Brain Injury

To our knowledge the only therapeutic agents that have been explicitly trialed for neuroprotection in preterm infants are erythropoietin (PENUT trials) [150] and magnesium sulfate [122]. Magnesium sulfate has a drawback that treatment must be prophylactic, meaning the unnecessary treatment of many infants to achieve efficacy for those with injury. Moreover, magnesium only appears to improve motor issues. However, retrospective analyses of caffeine administered to preterm infants for respiratory stimulation suggest it may offer neuroprotection [151,152,153,154,155]. One of the most well-known prospective studies—caffeine for the treatment of apnea (“CAP”)—randomized a large sample of preterm infants to caffeine or placebo treatment for respiratory stimulation. Results showed that preterm infants treated with caffeine citrate (20 mg/kg loading dose; 5–10 mg/kg daily maintenance) had better survival without neurodevelopmental disability, and lower rates of CP and cognitive delay at 18–21 months [156]. By 5 years of age caffeine was still associated with enhanced motor function, but not reduced rates of cerebral palsy or intellectual impairment [157]. However, other recent studies continue to support beneficial outcomes from caffeine, based primarily on retrospective assessment of caffeine administered for respiratory applications in preterm infants. Moreover, studies show that benefits from early/immediate caffeine treatment are more robust than later caffeine administration [151,152,153,154,155].

These clinical findings are consistent with animal work showing that treatment with caffeine citrate (50 mg/kg/day) from postnatal day 1 (P1) to P12 increased pyramidal neuronal growth in prefrontal cortex of rats at P35 and P70 [158]. Such growth-promoting effects could explain improved cognitive function reported in both children and rats treated with caffeine after brain injury. In another study, caffeine citrate (15–20 mg/kg/day) administered to rats from P2 to P6 reduced seizure susceptibility to some chemo-convulsants in both juvenile and adult rats [159]. Further, rat pups exposed to caffeine from P0–P12 or P1–7 via treated lactating dams and then subjected to induced injury showed enhanced myelination, with reduced ventriculomegaly and tissue loss relative to untreated injured pups [160,161]. Mice with neonatal HI were also shown to benefit from a single dose of caffeine [162]. These combined findings support rigorous testing of the therapeutic benefits of caffeine in preterm HI injury rat models.

Our lab conducted two published studies using caffeine treatment in moderate to late-preterm neonatal rat models of HI injury [149,163] and found a consistent benefit of caffeine treatment following HI. In the first study, we assessed effects of one-time caffeine in P7 HI male rats (late preterm), using a Rice-Vannucci injury (120 min hypoxia). Caffeine was administered immediately after injury. We found beneficial long-term effects of caffeine treatment on several tasks, including significant improvement on the Morris Water Maze task [93]. We also saw decreased injury-related brain pathology in caffeine-treated HI rats relative to untreated HI rats in adulthood. In the second study, we assessed the effects of an immediate plus 24 h-delayed dose of caffeine in male rats with HI induced on P6 [149]. Again, we found significant benefits of caffeine treatment on a Rotarod task, Silent Gap 0–100 ms acoustic detection task, and Non-Spatial Water Maze (though trends were seen on other tasks). We also found reduced injury-related pathology in caffeine-treated HI compared to untreated HI rats in adulthood. Importantly, a recent pilot study extended these beneficial effects to P6 HI-injured female rats, with improved performance on Silent Gap 0–100 ms and Morris Water Maze, and reduced neuropathologic indices in caffeine-treated P6 HI female rats compared to HI-saline female littermates [164].

**Figure 2:**
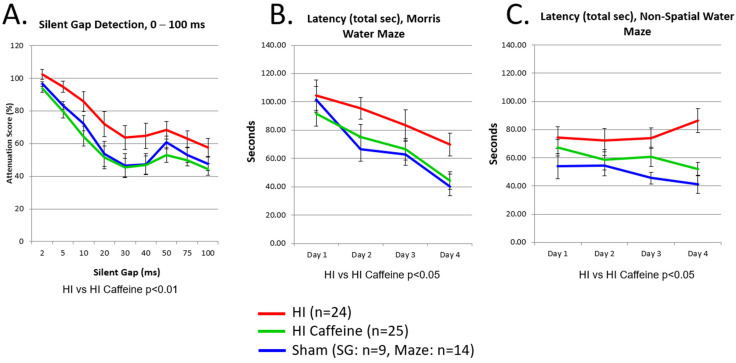
Caffeine Effects (Males). Performance in HI saline-treated (red), HI caffeine-treated (green) and sham-saline (blue) male rats on: (**A**) Silent Gap 0–100 ms detection; **(B**) spatial Morris Water Maze (MWM); and (**C**) Non-spatial water maze (NSM). Unpublished data re-analysis Adapted with permission from Refs. [93,149]. 2013 by the authors and 2018 by ISDN.

We performed a *post hoc* pooled multi-variate ANOVA to test for differences between P6 and P7 rats. This also included analysis of a single vs. multiple dose regimen of therapy. Sex was not use as a variable. Results of a repeated measures ANOVA showed robust significant benefits of caffeine on outcome measures. We found no difference in the beneficial effect between P6 and P7. We continued to see superior behavioral effects with caffeine treated pups compared to non-treated on Silent Gap acoustic discrimination 0–100 ms (F(1,47) = 11.3, *p* < 0.01; Figure 2A); Morris Water Maze (F(1,47) = 4.2, *p* < 0.05; Figure 2B); and Non-spatial Water Maze (F(1,47) = 4.5, *p* < 0.05; Figure 2C). In all analyses, Day was significant (reflecting ongoing improvement) but did not interact with Treatment. Task was further included as a repeated-measures variable (3 levels), and here the effect size for the difference between HI saline and HI caffeine-treated rats was quite robust (0.93 effect size; F(1,47) = 10.5, *p* < 0.01), confirming that these three tasks together (SG-100, MWM and NSM) provide an excellent and sensitive tool for preliminary screening of therapeutic caffeine treatment parameters in a rat neonatal HI model.

## 5. Mechanisms of Hypoxia/Ischemia, Cell Death, and Brain Injury of Prematurity

As mentioned in the introduction, the exact mechanisms of action of hypothermia and caffeine treatment are not fully understood. For hypothermia, it is thought that benefits are seen through preservation of energy metabolism and a reduction in cytotoxic edema, free radicals, inflammation, and apoptotic cell death. Caffeine, as a non-selective adenosine antagonist is thought to act at the A_1_ and A_2A_ receptors to reduce calcium influx, and potentially reduce microglial activation or the release of inflammatory compounds [15,16,17].

We recently addressed this question in a study examining the effects of caffeine treatment following P6 HI injury in a rat model on microglial activation as measured 48 h post-injury. Both male and female rats were assigned to either a P6 Sham, HI injury followed by saline, or HI injury followed by caffeine treatment [165]. Results showed that both male and female rats with P6 HI injury and saline treatment exhibited significantly elevated chromatin condensation 48 h after injury in the right cortex (side of HI injury) as measured by concentrated DAPI staining. Male and female rats with P6 HI injury followed by caffeine treatment did not differ significantly from shams and had chromatin condensation values mid-way between sham and HI saline animals. The lack of significant chromatin condensation in HI caffeine rats relative to shams—despite a robust effect in HI rats treated only with saline, confirms behavioral evidence of therapeutic caffeine effects, as observed using behavioral measures shown above [165]. Interestingly, though we saw similar reductions in cell death between the sexes, when we looked at microglial activation as measured by soma-size of Iba-labelled cells, we saw trending microglial activation differences in HI saline male rats versus HI caffeine male rats (*p* = 0.08, one-tail) specifically with less activation in caffeine treated subjects. A very different pattern was seen in females, where we saw no significant microglial activation in HI saline animals relative to shams, nor effects of caffeine on microglial activation. These results suggest that therapeutic effects of caffeine in neonatal males may occur at least in part via reductions in microglial activation, whereas female benefits from caffeine appear to be conferred via different protective mechanisms. This is despite the fact that, both sexes show structural and functional benefits following caffeine treatment (per cumulative studies).

## 6. Sex Differences in Neonatal HI and Therapeutic Intervention

Our overall findings combined with the existing literature highlight the importance of including sex differences in studies of perinatal brain injury in term and preterm infants as well as sex differences in response to therapeutic intervention [128,161,166,167,168]. While caffeine appears to exert similar levels of protection in both sexes, hypothermia appears to have different degrees of benefit in males and females [104,164]. Surprisingly few neonatal brain injury outcome studies consider sex as a variable, and none of the human trials provide outcome data for males and females separately. Yet, human research supports differences in the events surrounding HI in males and females. Male infants are 61% more likely to experience brain injury [169,170], and have a higher incidence of prematurity, anoxia, intraventricular hemorrhage, and mortality from prematurity [171,172,173,174,175]. Males are more likely to be diagnosed with developmental disorders including speech and language disorders and ADHD. Males develop CP at a higher frequency relative to females with similar brain injury [171,176,177,178,179]. The heightened susceptibility of males to HI insult is coupled with exacerbated cognitive/behavioral deficits following HI [48,173,180,181,182,183].Preterm males either with or without with neonatal HI injuries score lower than comparable preterm females on cognitive and developmental outcomes [38,39,43,49,171,175,176,180,184,185]. These differences were summarized in a meta-analysis performed by our lab showing significantly worse IQ outcomes for preterm male infants compared to matched females [91]. Despite important clinical implications, precise mechanisms underlying sex differences in outcomes are not well understood. Testosterone may exacerbate neural injury in the male or estrogen/progesterone could be protective in the female [88,140,186,187,188,189]. Other evidence suggests sex differences in cell death pathways may favor females [10,100,190,191,192,193] as well as sex differences in inflammation, microglial activation, and/or post-injury cell genesis [189,194,195]. Knowledge about the mechanisms of this female advantage could lead to novel therapeutic discovery. Moreover, males and females may respond differently to interventions, which is clinically important [128,166,167,168,176,180]. A classic illustration is the case of indomethacin, which was widely used in preterm infants to lower IVH risk, and later discovered to be effective only in males [196,197].

Overall results show that although caffeine reduces cell death in both male and female rats as measured by DAPI to index chromatin condensation, offering equivalent protection, the intermediary impact on microglial activation as a therapeutic mechanism may be quite different in the sexes. This could have critical implications for individual optimization of timing, dosing, and/or interactions between caffeine and other adjunct interventions in at-risk male versus female infants. Our results may also indicate that different mechanisms of cell death in neonatal males and females following HI may lend themselves to sex-specific next generation interventions.

## 7. Conclusions

Translational work and animal models looking at preterm HI are extremely important especially because preterm infants are at higher risk of brain injury and there are no approved treatments. This contrasts term infants, where injury risk is lower but approved treatment exists. It also appears from work in our lab and others that what is therapeutic for term infants may not be effective for preterm infants (e.g., hypothermic treatment) or what works/is beneficial for one sex may not be for the other (indomethacin, mild hypothermia, and caffeine). In addition, the effect of injury appears to have different outcomes in extremely preterm vs. mildly preterm infants [92]. Thus, extensive animal modeling helps to avoid later discoveries of adverse outcomes once treatment trials move to humans.

Current work from our lab and other studies show that mild hypothermia and caffeine treatment have great potential to be effective treatments for preterm HI. There are other potential treatments in the translational pipeline, such as metformin, exendin-4, and leptin [198,199,200]. Further work on potential treatment must model conditions of prematurity and sex. As seen above, sex and gestational age may affect the mechanism of protection as well as how effective the treatment is. Modeling injury in rodents < P7, reporting sex differences, and looking at a wider variety of induced brain injury (because there are many potential causes of brain injury in the preterm), would help to ensure we can better transition from pre-clinical animal models into human trials. To further help validate our models, there are several factors that may contribute to development of preterm brain injury that should also be considered (genetics, inflammatory conditions, other comorbidities, and recurrent hypoxia). These other variables must be included and addressed in research to ensure we are using the most valid modeling we can, which will enhance the transition into human studies.
